# Occurrence of foodborne pathogens in Italian soft artisanal cheeses displaying different intra- and inter-batch variability of physicochemical and microbiological parameters

**DOI:** 10.3389/fmicb.2022.959648

**Published:** 2022-08-25

**Authors:** Frédérique Pasquali, Antonio Valero, Arícia Possas, Alex Lucchi, Cecilia Crippa, Lucia Gambi, Gerardo Manfreda, Alessandra De Cesare

**Affiliations:** ^1^Department of Agricultural and Food Sciences, Alma Mater Studiorum - University of Bologna, Bologna, Italy; ^2^Department of Food Science and Technology, University of Córdoba, Agrifood Campus of International Excellence ceiA3, Córdoba, Spain; ^3^Department of Veterinary Medical Sciences, Alma Mater Studiorum - University of Bologna, Bologna, Italy

**Keywords:** microbiological quality, bacterial pathogens, soft cheese, processing environment, artisanal production

## Abstract

Artisanal cheeses are produced in small-scale production plants, where the lack of full automation and control of environmental and processing parameters suggests a potential risk of microbial contamination. The aim of this study was to perform a longitudinal survey in an Italian artisanal factory producing a spreadable soft cheese with no rind to evaluate the inter- and intra-batch variability of physicochemical and microbial parameters on a total of 720 environmental and cheese samples. Specifically on cheese samples, the evaluation was additionally performed on physicochemical parameters. Cheese samples were additionally collected during 15 days of storage at constant temperatures of 2 and 8°C, as well as a dynamic profile of 2°C for 5 days and 8°C for 10 days. Furthermore, Enterobacteriaceae isolates were identified at species level to have a better knowledge of the environmental and cheese microbiota potentially harboring human pathogens. High inter-batch variability was observed for lactic acid bacteria (LAB) and total bacteria count (TBC) in cheese at the end of production but not for pH and water activity. A temperature of 8°C was associated with a significantly higher load of Enterobacteriaceae in cheeses belonging to batch 6 at the end of storage, and this temperature also corresponded with the highest increase in LAB and TBC loads over cheese shelf life. Results from generalized linear mixed models (GLMMs) indicated that drains in the warm room and the packaging area were associated with higher levels of TBC and Enterobacteriaceae in cheese. Regarding foodborne pathogens, no sample was positive for verotoxigenic *Escherichia coli* (VTEC) or *Listeria monocytogenes*, whereas six *Staphylococcus aureus* and one *Salmonella* pullorum isolates were collected in cheese samples during storage and processing, respectively. Regarding Enterobacteriaceae, 166 isolates were identified at species level from all batches, with most isolates belonging to *Klebsiella oxytoca* and *pneumoniae*, *Enterobacter cloacae*, *Hafnia alvei*, and *Citrobacter freundii* evidencing the need to focus on standardizing the microbial quality of cow milk and on hygienic procedures for cleaning and disinfection especially in warm and maturation rooms. Further studies should be performed to investigate the potential pathogenicity and antimicrobial resistance of the identified Enterobacteriaceae species in artisanal cheeses.

## Introduction

Artisanal foods are increasing in popularity since they are perceived as homemade and more genuine products (Cirne et al., 2019). Moreover, these products usually benefit from an undeniable and recognized richness of taste, providing valuable organoleptical properties which are highly appreciated by consumers. The practices or technologies applied and the know-how of the artisanal food producers are protected from imitation and misuse by the European Union (EU) geographical indication schemes such as Protected Designation of Origin (PDO) (European Union [EU], 2012). As regards quality standards, Italy is the EU country with the highest number of PDOs (European Commission [EC], 2022).

These quality standards are also linked to various biodiversity factors, such as microbial communities brought by ingredients and production environments. Due to the lack of standardization in the artisanal productions, data on the microbial parameters of these products are scarce.

As artisanal cheeses are produced in small-scale production plants where control of the process is often challenging due to the lack of full automation and control of environmental parameters and processing variables, there is a suggested potential risk of microbial contamination and growth (Jaramillo-Bedoya et al., 2021). In this regard, it is important to monitor the microbial quality of the food and the environment to reduce the exposure of consumers to microbial hazards. In the dairy industry, standard microbial testing focuses on the enumeration of total mesophilic bacteria, coagulase-positive staphylococci and Enterobacteriaceae as contamination indicators (Fusco et al., 2020). The presence of Enterobacteriaceae, although recognized as part of the natural microbiota of many cheeses, in high concentrations is usually indicative of poor microbiological quality (Maifreni et al., 2013).

Microbial testing is particularly relevant in those cheeses posing a higher risk for consumers, namely, those which have a short maturation time and with a higher water content (> 45%) (named “soft”), with no rind, and a short shelf life (e.g., < 15 days). Italy has a long tradition of artisanal soft cheeses with PDO; Squacquerone di Romagna, Casatella Trevigiana, and Robiola di Roccaverano are some examples. For soft cheese made with pasteurized cows’ milk, according to EU regulations No. 853/2004 and No. 2073/2005, microbiological analyses by food business operators should focus on raw cows’ milk and the final cheese product (European Union [EU], 2004, 2007). Processed cows’ milk cannot exceed a load of 100,000 cfu/mL at 30°C when directly used to produce dairy products (European Union [EU], 2004). In soft cheese at the end of the manufacturing process, coagulase-positive staphylococci cannot exceed 100 cfu/g in more than two over five samples tested.

From a food safety perspective, soft cheese can be a vehicle of different microbial hazards. Because of its tolerance to a wide range of environmental conditions, *Listeria monocytogenes* can grow and survive in the processing plant and contaminate food during production. *L. monocytogenes* outbreaks associated with the consumption of contaminated fresh and/or soft cheeses have been reported worldwide, including the United States, Sweden, Canada, Czechia, Austria, Portugal among other countries (Jackson et al., 2018; Martinez-Rios and Dalgaard, 2018). *Staphylococcus aureus* often causes mastitis in cows, leading to milk contamination (Rabello et al., 2007). It also inhabits the skin and nasal cavity of food handlers, who might act as vehicles of transmission of virulent strains to food (Castro et al., 2016). Outbreaks of *S. aureus* have been associated with the consumption of contaminated milk or cheese (Johler et al., 2015a,b). In Italy, *S. aureus* has been isolated from raw milk and artisanal raw milk cheeses (Johler et al., 2018). *Salmonella* spp. has also been a common causative agent of foodborne outbreaks linked to cheese products being contaminated with microbes associated with the primary production and processing environment (Robinson et al., 2018). Regarding *Escherichia coli*, Shiga toxin-producing *E. coli* (STEC) was associated to outbreaks related to the consumption of raw milk and raw milk cheeses in the United States in 2009–2014 and in France in 2019 (Costard et al., 2017; Jones et al., 2019). Although the pathogen is inactivated by pasteurization, STEC can be detected in the final product as a result of post-process contamination (Dos Santos Rosario et al., 2021).

Based on EU regulations, when process hygiene criteria are not fulfilled, corrective measures are required during cheesemaking operations. Therefore, knowledge on microbial loads at each stage of production is of paramount importance to select specific and suitable control measures in real time. The microbiological status at each stage of production of both the food product and the environment could be helpful to describe the whole food production chain and to specifically evaluate the intra- and inter-batch variability of artisanal productions. At present, very few scientific papers have focused on the microbial quality of soft cheese made from raw or pasteurized cow or goat cheese worldwide (Pappa et al., 2017; Barukèiæ et al., 2020; Silva et al., 2021), and none focused on artisanal PDO products.

The aim of this study was to perform a longitudinal survey in an Italian artisanal factory producing a spreadable soft cheese with no rind to evaluate the physicochemical and microbial parameters during the different production stages. Quantitative data collected over a year were analyzed through statistical models to identify the intra- and inter-batch variability of total bacterial count (TBC), Enterobacteriaceae, and lactic acid bacteria (LAB) from raw materials, environmental and product samples. Among these data, potential correlations within different stages of the production chain were investigated. Moreover, a durability study was carried out to investigate the microbial fate of artisanal cheeses during 15 days at constant temperatures of 2 and 8°C, as well as a dynamic profile of 2°C for 5 days and 8°C for 10 days. Furthermore, Enterobacteriaceae isolates were identified at species level to have a better knowledge of the environmental and cheese microbiota.

## Materials and methods

### Experimental design

The cheese production chain of one Italian artisanal factory was sampled over 14 months (January 2020–March 2021). The factory is producing a spreadable soft cheese with no rind made of pasteurized cow milk. Regarding processing, after milk pasteurization, starter cultures, calf rennet, and enzymes were added to the milk. After coagulation and clot disruption, curd was drained. After storage in the warm room at 25°C for 1.5 h, cheese was ripened at 5°C for 1–4 days in the maturation room, and then it was packed in the packaging area. Raw materials and intermediate and final products were sampled along with the swabs from the processing environment. A total of 720 samples were tested, namely, unpasteurized cow milk (*n* = 24), pasteurized milk (*n* = 24), calf rennet (*n* = 30), and cheese (*n* = 78) and environmental samples [walls (*n* = 56) and drains (*n* = 72)] from the warm room, the maturation room, and the packaging area. Additionally, workers’ gloves (*n* = 20) at the packaging area were sampled. Environmental samples were collected by swabbing a 100 cm^2^ area with a sterile cotton swab (Copan Italia, Brescia, Italy) that has been moistened in 10 mL of saline solution (0.9% NaCl). Environmental samples were collected before cleaning and disinfection procedures. Additionally, within a durability study, samples of soft cheese (*n* = 416) were analyzed at specific time intervals (i.e., 0, 1, 4, 8, 11, and 15 days) during storage at 2°C, 8°C, and dynamic temperatures (i.e., 2°C for the first 5 days and 8°C for the remaining 10 days of storage). Five sample units per matrix (i.e., food and environment) were tested for each batch. Overall, six batches were investigated: batch 1 (January 2020), batch 2 (May 2020), batch 3 (July 2020), batch 4 (November 2020), batch 5 (January 2021), and batch 6 (March 2021).

### Microbiological analyses

Total bacterial counts (TBCs) were enumerated in all samples collected at different production stages along with pH and water activity (a_w_) of cheese samples following international standard protocols (ISO, 1999, 2004a,^2013^). LAB (ISO, 1998) and Enterobacteriaceae (ISO, 2017a) were also quantified on raw materials and final products during shelf life lasting for 15 days. The occurrence of *L. monocytogenes* (ISO, 2017b), coagulase-positive Staphylococci (ISO, 2004b), verotoxigenic *E. coli* (VTEC) (using ISO protocols and subsequent PCR for detection of Shiga toxin genes) (ISO, 2001; Perelle et al., 2004), and *Salmonella* spp. (ISO, 2017c) was also investigated on cheese samples and environmental sites. Moreover, to isolate and identify bacteria belonging to the Enterobacteriaceae family, 25 g of cheese was diluted in 225 mL of Buffer Peptone Water (BPW, Thermo Scientific, Milan, Italy) and incubated for 24 h at 37°C. For Enterobacteriaceae species identification, BPW pre-enriched cultures were then streaked on MacConkey agar (Thermo Scientific) and incubated for 24 h at 37°C. Five colonies per plate were harvested and submitted for confirmation by biochemical test (RapID ONE System and RapID STAPH PLUS System, Thermo Scientific) and PCR (Wesley et al., 2002; Perelle et al., 2004; Chander et al., 2011; Saraiva et al., 2018). If confirmation was obtained, one isolate per species per sample was retained.

### Minimum inhibitory concentration

Since the relevance of multidrug-resistant *Klebsiella* spp. as human bacterial pathogen of nosocomial importance and since the number of *Klebsiella* spp. isolates was the most abundant within Enterobacteriaceae, the minimum inhibitory concentration of *Klebsiella oxytoca* and *K. pneumoniae* species was evaluated using Sensititre EUVSEC plates (Thermo Fisher Scientific Sensititre EU Surveillance Salmonella/E. coli EUVSEC Plate).

### Data analysis and modeling

Data analysis and modeling were performed using R (R Core Team, 2020). Microbial counts determined from different food batches and different environmental samples were statistically compared by ANOVA (*p* ≤ 0.05). The Tukey HSD test was performed to identify the different homogeneous groups. Boxplots and barplots were constructed to enable the visualization of intra- and inter-batch variability on microbial counts determined on cheese samples stored at different temperatures during their shelf life.

### Generalized linear mixed models

Generalized linear mixed models (GLMMs) were adjusted to longitudinal data sets from environmental surfaces, food contact surfaces, intermediate products, finished cheeses, and stored cheeses, including the estimation of inter-batch variability as random effect. To do so, the R packages, lme4 (Bates et al., 2015) and *nlme*, were used. Main effects considered the influence of the variables “Stage” (warm room, maturation room, packaging area, and storage) according to the form:


(1) $$Y_{i\,s\,\left(b\right)}=\left(\beta_{0}+u_{b}\right)+S\,t\,a\,g\,e_{s}+\varepsilon_{i\,s\,\left(b\right)}$$

where *Y*_*is(b)*_ is the count (log cfu/g) of a given microbial group (TBC, Enterobacteriaceae, and LAB) determined in the processing stage *s*, belonging to batch *b*; β*_0_* is the model intercept that can take random shifts *u*_*b*_ according to batch *b*; *Stage*_*s*_ is the processing stage *s;* and ε*_*is(b)*_* is the error of the microbial count *i* determined in the processing stage *s*, belonging to batch *b*.

Samples (environment and drains swabs, workers’ gloves, intermediate cheese product, finished cheese, and stored cheese) taken from each processing stage were included in another main effects model as follows:


(2) $$Y_{i\,s\,\left(b\right)}=\left(\beta_{0}+u_{b}\right)+S\,t\,a\,g\,e_{s}\,\left(E\,n\,v\,i\,r\,o\,n\,m\,e\,n\,t_{i}\right)+\varepsilon_{i\,s\,\left(b\right)}$$

where *Stage_*s*_ (Environment_*i*_)* is the sample *i* taken from the processing stage *s* belonging to batch *b*.

The between-batch variability (within a factory) was determined from the squared standard deviation of the normal distribution for the random effects. Errors are also assumed to follow a normal distribution.

## Results and discussion

### Enumeration of total bacterial count, lactic acid bacteria, and Enterobacteriaceae

**FIGURE 1:**
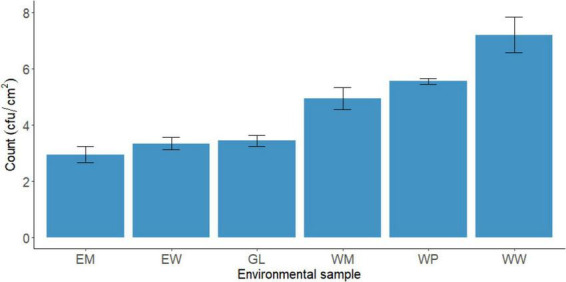
Total bacterial count (TBC) on environmental samples collected at the processing plant. EM, walls, maturation room; EW, walls, warm room; GL, gloves of workers, packaging area; WM, drains, maturation room; WP, drains, packaging area; WW, drains, warm room (mean ± standard deviation of six batches).

As regards environmental swabs collected at the processing plant, statistical differences were recorded on TBC within the different types of samples (*p* ≤ 0.05). Drains samples presented the highest loads of TBC compared to the other tested environmental sites. More specifically, drains samples of the warm room (WW) showed the highest TBC with a mean value of 7.07 ± 1.10 log cfu/cm^2^, followed by drains of the packaging area (WP, mean value 5.43 ± 0.94 log cfu/cm^2^) and drains of the maturation room (WM, mean value 4.83 ± 1.02 log cfu/cm^2^) (Figure 1).

As regards milk samples, statistical differences in TBC were detected between the investigated batches (*p* ≤ 0.05), with batch 2 and batch 6 showing the highest and the lowest TBC values, respectively (Table 1). Pasteurization (T ≥ 72°C for 20 s, 6,000 L/h) led to a decrease of microbial loads of 3 log reductions on average. In particular, TBC decreased from 6.48 ± 0.49 to 3.34 ± 0.76 log cfu/mL. The levels of TBC in milk after pasteurization were compliant with the requirements of EU regulation 853/2004 for processed cows’ milk intended for the elaboration of dairy products (TBC no higher than 100,000 cfu/mL) (European Union [EU], 2004).

**TABLE 1:** Enumeration of total bacterial count, lactic acid bacteria, and Enterobacteriaceae in raw materials and cheese during processing.

Sample*	Batch 1	Batch 2	Batch 3	Batch 4	Batch 5	Batch 6
**Total bacterial count**						
Milk before pasteurization	6.24 ± 0.57^ac^**	7.37 ± 0.13^b^	6.28 ± 0.22^ac^	6.46 ± 0.19^a^	6.56 ± 0.11^a^	5.94 ± 0.11^c^
Milk after pasteurization	2.65 ± 0.25^a^	4.16 ± 0.09^b^	3.52 ± 0.13^ab^	3.15 ± 0.12^ab^	2.36 ± 0.30^a^	4.18 ± 1.38^bc^
Calf rennet	2.28 ± 0.36^ac^	3.09 ± 0.20^b^	2.00 ± 0.00^a^	2.30 ± 0.30^ac^	2.76 ± 0.56^ab^	3.16 ± 0.54^b^
Cheese in the warm room	3.90 ± 0.17^a^	4.02 ± 0.20^a^	5.03 ± 0.09^b^	5.08 ± 0.18^b^	5.27 ± 0.07^bc^	5.49 ± 0.10^c^
Cheese in the maturation room	3.32 ± 0.20^a^	3.69 ± 0.19^b^	5.59 ± 0.09^c^	4.46 ± 0.19*^d^*	5.87 ± 0.05^c^	5.80 ± 0.06^c^
Packed cheese in the packaging area	3.53 ± 0.07^a^	4.54 ± 0.19^b^	5.63 ± 0.06^c^	4.73 ± 0.56^b^	5.90 ± 0.16^c^	5.67 ± 0.03^c^
**Lactic acid bacteria**						
Cheese in the warm room	2.28 ± 0.63 (2)***	2.65 ± 0.18 (2)	2.50 ± 0.82	1.18 ± 0.32 (2)	1.72 ± 0.00 (4)	1.33 ± 0.10 (2)
Cheese in the maturation room	1.70 ± 0.00 (4)	1.35 ± 0.20 (2)	1.67 ± 0.19	3.71 ± 0.02	1.43 ± 0.41 (1)	2.08 ± 0.46
Packed cheese in the packaging area	1.96 ± 0.24	1.48 ± 0.00 (4)	1.64 ± 0.27	3.69 ± 0.09	1.77 ± 0.15 (2)	2.30 ± 0.59
**Enterobacteriaceae**						
Cheese in the warm room	1.40 ± 0.00 (4)	<1.00 ± 0.00	1.81 ± 0.00 (4)	<1.00 ± 0.00	<1.00 ± 0.00	<1.00 ± 0.00
Cheese in the maturation room	<1.00 ± 0.00	1.40 ± 0.32 (3)	<1.00 ± 0.00	<1.00 ± 0.00	<1.00 ± 0.00	<1.00 ± 0.00
Packed cheese in the packaging area	<1.00 ± 0.00	<1.00 ± 0.00	<1.00 ± 0.00	<1.00 ± 0.00	<1.00 ± 0.00	1.86 ± 0.15 (2)

*Microbial counts in cheese samples are expressed in log cfu/g, while for raw materials (milk and calf rennet), they are expressed as log cfu/Ml.

**Different superscript letters indicate significant differences in microbial counts between batches (*p* ≤ 0.05).

***Number of samples with values under the detection limit excluded from the calculation of the mean.

**FIGURE 2:**
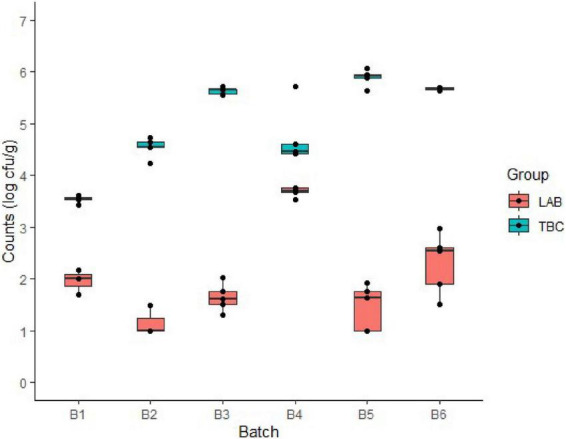
Lactic acid bacteria (LAB) and total bacterial counts (TBCs) in packaged cheese samples belonging to the six tested batches (B) at the end of the production process.

For the soft cheese samples collected at different stages of the production process, the TBC counts were constant during the production stages with no differences when compared to cheese sampled in the warm, maturation, and packaging rooms in each batch. Low intra-batch variability was observed in these samples (Table 1). However, inter-batch variability was observed with average differences of TBC up to 2.4 log cfu/g [i.e., 3.53 (batch 1) vs. 5.90 (batch 5) log cfu/g quantified on packed cheese collected at the packaging area] with batches 3, 5, and 6 showing the highest loads (Figure 2). Inter-batch variability was observed for LAB especially in packed cheese [i.e., 1.06 (batch 2) vs. 3.69 (batch 4) log cfu/g] with batch 4 showing the highest load (Figure 2). Moreover, LAB counts showed higher intra-batch variability compared to TBC especially in batches 5 and 6 (Figure 2). At the end of processing, LAB showed higher counts in batch 4 in comparison with the other batches (Figure 2).

The high variability of LAB counts might be due to the fat composition of milk. In summer the fat composition of milk is lower than in winter (summer fat content 3.70–3.80%, winter fat content approximately 4.10–4.20%) (unpublished data). Although it is clear that the fat content plays a role, different effects are reported on different starter cultures and non-starter culture Lactobacilli in dairy products. Yogurt with a lower fat content was already described as associated with higher growth capability of *Lactobacillus acidophilus* (Tavakoli et al., 2019). Similarly, high LAB content was observed in low-fat feta cheese at the end of 14 days storage at 4°C (Ahmed et al., 2021). In contrast, non-starter culture *Lactobacillus casei* showed higher growth performances with higher fat content in cheddar cheese (Tan et al., 2012).

In the packed cheese collected at the packaging area, Enterobacteriaceae loads were below the detection limit of 1 log_10_ cfu/g in all samples tested from batches 1–5, whereas in batch 6 the mean Enterobacteriaceae concentration was 1.86 ± 0.15 log cfu/g (Table 1).

**FIGURE 3:**
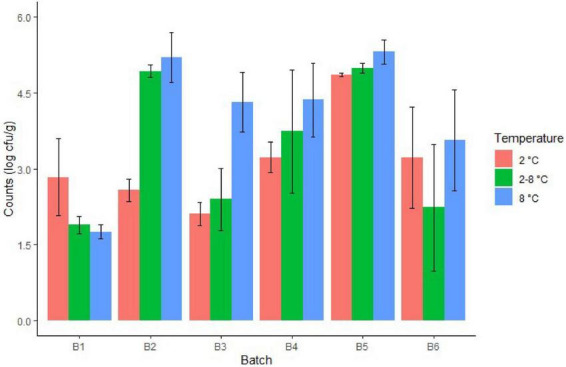
Lactic acid bacteria count in cheeses from different batches after 15 days of storage at different temperatures.

**FIGURE 4:**
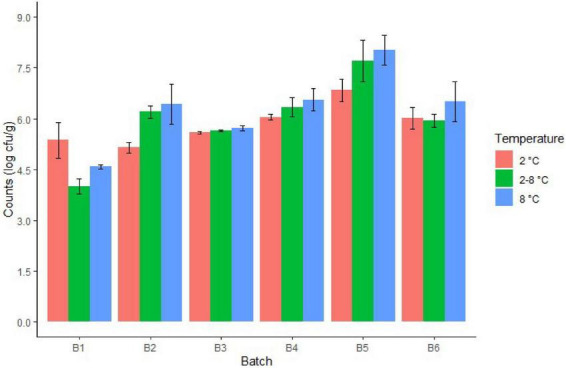
Total bacteria counts in cheeses from different batches after 15 days of storage at different temperatures.

Regarding cheese stored at different temperatures, the fate of TBC and LAB showed an increase over cheese shelf life, although storage at 2°C was associated to a slower increase (Table 2). Specifically, approx. 4–5 log cfu/g at day 8 at 8°C (batch 4), at day 11 at 2/8°C (batches 2, 4, and 5) and at day 15 at 2°C (batch 5) (Figure 2). Regarding inter-batch variability, after 15 days of storage, differences of up to approximately 2 log cfu/g at 2°C and 3 log cfu/g at 8 and 2/8°C were observed for LAB (Figure 3). Similar inter-batch variability was observed at the same day of sampling for TBC with differences of approximately 2.5–3.5 log cfu/g at 8°C and 2/8°C. However, no differences were observed at 2°C (Figure 4). Both bacterial groups showed the highest concentrations in batch 5, reaching 5.31 and 8.03 log cfu/g, respectively, for LAB and TBC after 15 days of storage at 8°C (Figures 3, 4).

**TABLE 2:** Mean increase of microbial concentrations on Italian soft cheeses during 15 days of storage at different temperatures.

Temperature (°C)*	Total bacteria (log cfu/g)	Enterobacteria (log cfu/g)	Lactic acid bacteria (log cfu/g)
2	1.04 ± 1.00**	0.09 ± 0.22	1.58 ± 1.00
8	1.50 ± 1.04	0.73 + 0.90	2.57 ± 1.49
2–8	1.17 ± 1.13	0.39 ± 0.37	1.81 ± 1.61

*2–8 refers to 5 days at 2 °C followed by 10 days at 8 °C.

**Mean ± standard deviation for samples from 6 different batches.

Of all the investigated batches, batch 6 showed the highest intra-batch variability for LAB after 15 days of storage at all three tested temperatures (Figure 3). Reduced intra-batch variability was observed for TBC in cheese at the end of storage, whereas high variability was observed in batches 5 and 6 at the end of production (Figures 2, 4).

The counts of Enterobacteriaceae were under or close to the detection limit in all batches for all cheese samples during storage, except for batch 6 at day 15 at 8°C where a concentration of 3.06 ± 1.17 log cfu/g was observed (data not shown). Although not statistically significant, this result might be linked to a lower efficiency of the pasteurization process which was associated to a lower TBC reduction (1.76 log cfu/mL reduction in batch 6 in comparison with 3 log cfu/mL reduction on average in the other batches) (Table 1). A high coefficient of correlation has been previously observed between TBC and coliforms counts in bulk milk (Pantoja et al., 2009).

Temperature of 8°C is confirmed as an abuse temperature and associated in batch 6 with a significantly higher load of Enterobacteriaceae in the cheese at the end of production and the end of storage. In addition, the highest increase in LAB and TBC loads was recorded during cheese storage at this temperature (Table 2). In the literature, 8°C was determined as the lowest temperature that allows the growth of *Salmonella* in queso fresco (Kasrazadeh and Genigeorgis, 1994). In soft cheese, coliforms showed a higher increase rate during storage at 18°C in comparison with 4°C (Mohammed et al., 2019).

Moreover, no statistical differences were detected between the growth potential of a given microbial group at the different temperatures evaluated during cheese storage throughout its shelf life.

### Evaluation of physicochemical parameters (pH and a_w_)

**FIGURE 5:**
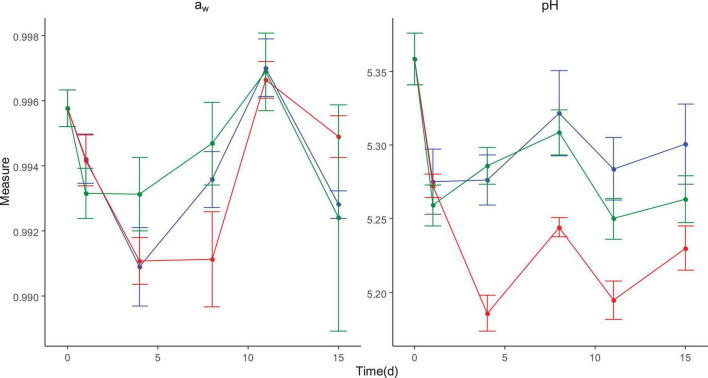
Mean ± standard deviation of a_w_ and pH of cheeses from all batches during storage for 15 days at 2°C (blue line), 2–8°C (green line), and 8°C (red line).

As regards raw materials such as milk, calf rennet, and cheese during production, as expected the pH decreased after the inclusion of the starter culture (*Streptococcus thermophilus*) (Table 3). However, only in batch 3 did the pH reach a value lower than 5.3 in packed cheese immediately at the end of production. This value is within the range of 4.9–5.3 reported for similar types of cheeses (Lante et al., 2006). During storage, the higher the temperature the lower the pH in line with the higher LAB count at 8°C (Figures 3, 5). As expected, a_w_ of cheese at 15 days of storage was lower than that of cheese at day 0 (end of processing) (Figure 5).

**TABLE 3:** pH and water activity (a_w_) of raw materials and Italian soft cheese during the production process.

Sample	pH	a_w_
Milk before pasteurization	6.850 ± 0.010*	0.997 ± 0.001
Milk after pasteurization	6.771 ± 0.014	0.998 ± 0.001
Calf rennet	5.368 ± 0.010	0.885 ± 0.001
Cheese at the warm room	5.828 ± 0.026	0.992 ± 0.002
Cheese at the maturation room	5.338 ± 0.048	0.995 ± 0.001
Cheese at the packaging area	5.359 ± 0.018	0.996 ± 0.001

*Mean ± standard deviation are provided.

For the cheese samples at the packaging area, pH and a_w_ did not show significant differences during production between batches with pH values ranging from a minimum of 5.24 to a maximum of 5.47 and a_w_ values ranging from 0.992 to 0.999.

### Generalized linear mixed models

GLMMs were developed to investigate whether the production stage and the environment could partially explain the inter-batch differences in TBC and Enterobacteriaceae counts in cheeses. The results of this analysis are shown in Table 4.

**TABLE 4:** Parameter estimates of the linear mixed models with random effects assessing the variables “Stage” and “Environment” as main effects on total bacterial counts (TBCs) and Enterobacteriaceae counts in the artisanal soft cheese, as well as estimates of inter-batch variability.

		TBC		Enterobacteriaceae	
Model*	Parameters	Estimate (SE)	Pr > |t|	Estimate (SE)	Pr > |t|
**Main effects:**	**Random effects (σ)**				
**stage**	Batch in factory	0.507	–	0.299	–
	Residual	1.148	–	0.918	–
	
	**Fixed effects**				
	
	Intercept	5.065 (0.240)	<0.001	1.301 (0.156)	<0.001
	Maturation	–0.867 (0.171)	<0.001	–0.453 (0.137)	0.001
	Packaging	–0.407 (0.171)	0.018	–0.618 (0.137)	<0.001
	Storage	0.430 (0.133)	<0.001	–0.157 (0.106)	0.139

**Main effects:**	**Random effects (σ)**				
**environment**	Batch in factory	0.511	–	0.299	–
	Residual	0.907	–	0.882	–
	
	**Fixed effects**				
	
	Intercept	4.800 (0.267)	<0.001	1.001 (0.202)	<0.001
	Environmental_w**	–1.470 (0.234)	<0.001	–0.239 (0.228)	0.295
	Drains_w	2.269 (0.234)	<0.001	1.122 (0.228)	<0.001
	Environmental_m	–1.857 (0.234)	<0.001	–0.674 (0.228)	0.003
	Drains_p	0.744 (0.234)	0.002	–0.245 (0.228)	0.282
	Workers gloves	–1.367 (0.234)	<0.001	–0.670 (0.228)	0.003

*TBC and Enterobacteriaceae in cheese samples of the warm room were set as reference category. Only the statistically significant environmental effects are depicted in the Table.

**w, warm room; m, maturation room; p, packaging area.

Results from GLMMs indicated that the storage stage had a significant positive effect on TBC in cheeses (0.430 ± 0.133), while drains in the warm room (2.269 ± 0.234) and the packaging area (0.744 ± 0.234) were also associated with higher levels of TBC in the products. In addition, drains in the warm room also had a positive effect on Enterobacteriaceae loads in cheeses (1.122 ± 0.228). Regarding the inter-batch variability, random effects indicate a higher variability between batches with respect to TBC compared to Enterobacteriaceae.

### Occurrence of bacterial pathogens

Regarding isolation of bacterial pathogens, out of the 720 samples analyzed, *L. monocytogenes* and VTEC were not found. Six isolates were positive for *S. aureus* and one for *Salmonella* spp. Regarding Enterobacteriaceae, 166 isolates were identified at species level, with most isolates belonging to *Klebsiella oxytoca* and *K. pneumoniae* (44), *Enterobacter cloacae* (39), *Hafnia alvei* (26), and *Citrobacter freundii* (21) (Table 5). *Hafnia alvei* is generally described as commensal bacteria. However, in some circumstances, they have been reported as pathogenic (Günthard and Pennekamp, 1996). Bacteria were collected from cheese and swab environmental samples along production and during storage with a particular lower occurrence in batch 1. The latter showed a lower load of TBC from milk after pasteurization (2.65 log cfu/g) (Table 1), through packed cheese (3.53 log cfu/g) and cheese after 15 days of storage (5.37 log cfu/g at 2°C, 4.58 log cfu/g at 8°C, and 3.99 log cfu/g at 2/8°C) (Table 2).

**TABLE 5:** Results of the species identification from the 166 Enterobacteriaceae isolates in the six tested batches (B1–B6).

Species	B1	B2	B3	B4	B5	B6	Total
*Klebsiella oxytoca*		7	2	6	10	16	41
*Klebsiella pneumoniae*			1		1	1	3
*Enterobacter cloacae*		1	33	2	3		39
*Hafnia alvei*	5		1	2	16	2	26
*Citrobacter freundii*	3	10	2	1	2	3	21
*Escherichia coli*			7			1	8
*Raoultella planticola*	1			5		2	8
*Cedecea davisae*			3				3
*Kluyvera cryocrescens*					1	2	3
*Salmonella pullorum*						1	1
*Moellerella wisconsensis*		1					1
*Leclercia adecarboxylata*					1		1
Other (Enterobacteriales)				6	3	2	11
Total	9	19	49	22	37	30	166

*S. aureus* and *Salmonella* spp. were isolated from batches 2, 5, and 6. Specifically, six isolates of *S. aureus* were collected in cheese during storage at 2°C (batch 2), 8°C (batch 5), and 2/8°C (batch 6), and one isolate of *Salmonella* spp. was collected from cheese at the maturation room in batch 6 (Table 5). In 2020, *Salmonella* spp. maintained its position as the first causative agent of foodborne outbreaks in Europe (EFSA and ECDC (European Food Safety Authority and European Centre for Disease Prevention and Control)., 2021). In the last 20 years, serovars Enteritidis, Stanley, Typhimurium, Muenster, Montevideo, Dublin, and Newport were identified as causative agents of salmonellosis outbreaks associated with the consumption of contaminated cheese of different types (soft and hard, from cow, goat, and sheep milk mainly unpasteurized) in Italy, Switzerland, the Netherlands, and France (Pastore et al., 2008; Dominguez et al., 2009; van Cauteren et al., 2009; Van Duynhoven et al., 2009; Robinson et al., 2018; Ung et al., 2019; Napoleoni et al., 2021). Of relevance, in this study one isolate of *Salmonella* spp. was collected from cheese in the maturation room in batch 6. Although no further *Salmonella* spp. isolates were collected in cheese during storage, this finding evidence specific concerns for food safety.

*S. aureus* has been associated with bovine mastitis (Barkema et al., 2006). Raw cows’ milk might be a source of introduction of *S. aureus* within the dairy production chain. Food handlers might be another source since *S. aureus* is part of the common microbiota found on the skin and mucous membranes where it is predominantly methicillin-susceptible (Strommenger et al., 2018). In Italian artisanal cheeses, an occurrence of 80% has been recently described, although outbreaks due to the consumption of contaminated cheese are rarely reported (Johler et al., 2015a,^2018^). The occurrence of this pathogen was also described in this study: six isolates of *S. aureus* were collected from soft cheese of batches 5 and 6 during storage. Further investigations should be performed to evaluate the source of cheese contamination (i.e., milk, food handlers). Moreover, further characterizations of *S. aureus* enterotoxins and antimicrobial susceptibility are needed to assess the potential pathogenicity of collected isolates and their similarity to the methicillin-resistant *S. aureus* mainly found in hospitals.

Regarding Enterobacteriaceae, *Klebsiella* spp. isolates were the most abundant identified from all batches but one (batch 1) (Table 5). Batch 6 harbored the majority of *K. oxytoca* isolates (Table 6). Accordingly, batch 6 was characterized by the highest load of Enterobacteriaceae (reaching 3.06 log cfu/g at day 15 of storage at 8°C). Regarding the different steps of processing, all *K. oxytoca* isolates were collected in the warm and maturation rooms and the packaging area both in food and the environment (data not shown). Regarding storage of cheese samples, *Klebsiella* spp. was collected from day 1 to day 15 at all three tested temperatures. Abuse temperatures were not correlated to a higher occurrence of *Klebsiella* spp., indicating its ability to survive in soft cheese also at 2°C. *K. pneumoniae* and *K. oxytoca* have not been previously associated to foodborne-confirmed human cases. These species might cause bovine mastitis in dairy cows (Massé et al., 2020). These microorganisms are also found in soil environments and as saprophyte in the nasal cavity and in the intestinal tract. Pathogenic *K. pneumoniae* and *K. oxytoca* are often linked to nosocomial outbreaks of particular concern due to their multi-resistance character (Podschun and Ullmann, 1998). In this study, all isolates were resistant to sulfamethoxazole and all but one to ampicillin, and none was resistant to meropenem (Table 5). Both penicillins and sulfonamides are among the three antimicrobial agents most sold in Europe for food-producing animals [European Surveillance of Veterinary Antimicrobial Consumption (ESVAC), 2021]. The isolate collected from the environment of the warm room in batch 6 was resistant to sulfamethoxazole, ciprofloxacin, nalidixic acid, colistin, ampicillin, and gentamicin. The isolate collected from a cheese sample of batch 6 in the first day of storage at 2°C was resistant to sulfamethoxazole, colistin, and ampicillin.

**TABLE 6:** Distribution of MIC values in *Klebsiella* spp. isolates (vertical lines—clinical breakpoints).

	Concentration of antimicrobial agent (μg/mL)																		
	0.008	0.015	0.03	0.06	0.12	0.25	0.5	1	2	4	8	16	32	64	128	256	512	1,024	> 1,024
Sulfamethoxazole																	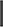		42*
Trimethoprim						27**	13	2			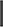								
Ciprofloxacin		18**	17	6			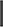	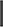 1											
Tetracycline									42**			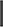							
Meropenem			38**	3				1											
Azithromycin											17	24	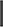 1						
Nalidixic acid										41**			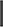		1				
Cefotaxime						41**		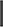	1	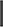									
Chloramphenicol											42**	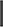							
Tigecycline						42**		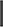											
Ceftazidime							42**		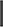		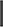								
Colistin								40**		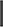 1			1*						
Ampicillin											1	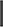	12	19	10*				
Gentamicin							30**	10	1	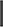 1									

*Number of isolates with MIC values higher than the concentration of the cell on the left.

**Number of isolates with MIC values lower or equal than the concentration of the cell. In white, the cells correspond to the tested dilution range.

In foods, *Klebsiella oxytoca* and *K. pneumoniae* were described as spoilage microorganisms of mozzarella cheese (Massa et al., 1992). In this study, 42 isolates of both species belonging to environmental and food samples were found, with a prevalence of 5.8% (42/720). *Klebsiella* isolates were selected after pre-enrichment of samples. This consideration can explain why they were isolated in batches in which the Enterobacteriaceae counts, performed without enrichment, were under the detection limit in all cheese samples collected at the packaging area and during storage. Although on a significantly lower number of samples (87), higher occurrences (43%) in dairy processing plants have been reported in Czechia by Gelbíčová et al. (2021), who isolated *Klebsiella* spp. in the processing environment, personnel, and raw material. None of the Czech strains were hypervirulent or multidrug-resistant. Further investigations should be performed on *Klebsiella* spp. isolates of this study, to evaluate the origin of cheese contamination (i.e., milk, personnel, processing environment), their antimicrobial susceptibility, and potential pathogenicity.

Similar to *Klebsiella* spp., *E. cloacae*, *Citrobacter freundii*, and *Hafnia alvei* were detected in cheese and environmental samples from the maturation room up to the end of processing and during storage. *E. cloacae* is widely encountered in nature. Similar to *Klebsiella* spp., some strains have been described as associated to mastitis in dairy cows and nosocomial infections of particular concern due to their resistance to antimicrobial agents including critical important ones of the highest priority (colistin, extended-spectrum cephalosporins, and carbapenems) (Schukken et al., 2012; Davin-Regli and Pagès, 2015). In foods, *E. cloacae* was collected from cheese and infant milk formulas (Maifreni et al., 2013; Amorim and Nascimento, 2017). In this study, 39 *E. cloacae* isolates were collected of which 33 were found in samples from batch 3. In this batch, *E. cloacae* was isolated during processing and storage at all three temperatures tested in cheese and environmental samples.

*H. alvei* and *C. freundii* are common inhabitants of the gastrointestinal tract, although, rarely, they have been associated to human infections. *H. alvei* was associated to respiratory tract infections (Ramos-Vivas, 2020), whereas *C. freundii* was associated to different human infections from urinary tract infections to bloodstream (Liu et al., 2018). In this study, 26 isolates of *H. alvei* and 21 of *C. freundii* were collected from batches 5 and 2, respectively. In the literature, *H. alvei* and *C. freundii* were isolated during cheese processing and ripening (Maifreni et al., 2013; Tabla et al., 2018).

## Conclusion

In conclusion, high inter-batch variability was observed among six batches of artisanal soft cheese production with batch 6 showing the highest Enterobacteriaceae counts at the end of the storage period of 15 days at 8°C. Isolation of *Salmonella*, *S. aureus*, *K. pneumoniae* and *K. oxytoca*, *E. cloacae, H. alvei*, and *C. freundii* after pre-enrichment in all batches during processing and storage suggests the need to focus attention on standardizing the processing steps from pasteurization to hygienic procedures. This includes cleaning and disinfection especially of warm and maturation rooms and the microbial quality and fat content of cows’ milk. Moreover, attention should be addressed to identify Enterobacteriaceae species occurring during processing and storage. Further studies should be performed to investigate the potential pathogenicity and antimicrobial resistance of the identified Enterobacteriaceae species in artisanal cheeses.

## Data availability statement

The original contributions presented in the study are included in the article/supplementary files, further inquiries can be directed to the corresponding author/s.

## Author contributions

FP contributed to data analysis, performed the literature search, and drafted the manuscript. AV and AP performed data and statistical analyses. CC and LG contributed to microbiological analyses. AL analyses of molecular biology. GM and AD contributed to data analysis. AD supervised the work. All authors contributed to data interpretation, manuscript revision and approved the final version as submitted.
